# The impact of the program for medical male circumcision on HIV in South Africa: analysis using three epidemiological models

**DOI:** 10.12688/gatesopenres.13220.1

**Published:** 2021-01-25

**Authors:** Eline L. Korenromp, Anna Bershteyn, Edina Mudimu, Renay Weiner, Collen Bonecwe, Dayanund Loykissoonlal, Clarence Manuhwa, Carel Pretorius, Yu Teng, John Stover, Leigh F. Johnson

**Affiliations:** 1 Center for Modeling and Analysis, Avenir Health, Geneva, Switzerland; 2 Department of Population and Health, NYU Langone Medical Center, New York, NY, 11016, USA; 3 Department of Decision Sciences, University of South Africa (UNISA), Pretoria, 0003, South Africa; 4 Research and Training for Health and Development, Johannesburg, 2196, South Africa; 5 National Department of Health, Pretoria, South Africa; 6 FHI 360, Pretoria, 0083, South Africa; 7 Independent Consultant, Pretoria, 0083, South Africa; 8 Center for Modeling and Analysis, Avenir Health, Glastonbury, CT, 06033, USA; 9 Centre for Infectious Disease Epidemiology and Research, University of Cape Town, Cape Town, South Africa

**Keywords:** HIV, prevention, program evaluation, mathematical modeling, health impact, cost-effectiveness

## Abstract

**Background**: South Africa began offering medical male circumcision (MMC) in 2010. We evaluated the current and future impact of this program to see if it is effective in preventing new HIV infections.

**Methods**: The Thembisa, Goals and Epidemiological Modeling Software (EMOD) HIV transmission models were calibrated to South Africa’s HIV epidemic, fitting to household survey data on HIV prevalence, risk behaviors, and proportions of men circumcised, and to programmatic data on intervention roll-out including program-reported MMCs over 2009-2017. We compared the actual program accomplishments through 2017 and program targets through 2021 with a counterfactual scenario of no MMC program.

**Results**: The MMC program averted 71,000-83,000 new HIV infections from 2010 to 2017. The future benefit of the circumcision already conducted will grow to 496,000-518,000 infections (6-7% of all new infections) by 2030. If program targets are met by 2021 the benefits will increase to 723,000-760,000 infections averted by 2030. The cost would be $1,070-1,220 per infection averted relative to no MMC. The savings from averted treatment needs would become larger than the costs of the MMC program around 2034-2039.

In the Thembisa model, when modelling South Africa’s 9 provinces individually, the 9-provinces-aggregate results were similar to those of the single national model. Across provinces, projected long-term impacts were largest in Free State, KwaZulu-Natal and Mpumalanga (23-27% reduction over 2017-2030), reflecting these provinces’ greater MMC scale-up.

**Conclusions**: MMC has already had a modest impact on HIV incidence in South Africa and can substantially impact South Africa’s HIV epidemic in the coming years.

## Abbreviations

ART = antiretroviral therapy; DHS = Demographic and Health Survey; HSRC = South Africa Human Sciences Research Council; (M)MC = (medical) male circumcision; PEPFAR = US President's Emergency Plan for AIDS Relief; PLWH = people living with HIV (PLWH); VLS = viral load suppression.

## Introduction

South Africa continues to face one of the highest burdens of HIV globally, despite extensive roll-out of prevention programs since the 1990s and treatment programs since 2004 ^1, 2^. In 2017, more than 20% of adults (15–49 years) were living with HIV (PLWH) in South Africa ^3^, with 56% on ART ^4^. Average national HIV prevalence among men and women reached 16% by 2002 ^5^, and has continued a slow rise to 18% by 2012 ^5– 9^ and 20% by 2017 ^3^ - in part an effect of PLWH surviving longer on ART, but in part also reflecting ongoing new HIV infections, which are declining only at a slow rate ^10^, highlighting concerns about sustainability of treatment programs.

Medical male circumcision (MMC) has been shown by three randomized control trials to reduce the transmission of HIV from females to males by about 60% ^11– 13^. It is a one-time intervention that provides life-long protection. ^14^. Recent studies have indicated that the risks of heterosexual transmission might be higher, 70–72% ^15, 16^, with less protection for male-to-male transmission risk of 20% ^16^. In response, the World Health Organization (WHO) and Joint United Nations Program on HIV/AIDS (UNAIDS) suggested a target of 80% MMC coverage by 2015 among adult men (ages 15–49 years) in 14 priority countries including South Africa ^17^.

South Africa adopted MMC strategy in 2009 and began service delivery in 2010. The 2012–2016 National Strategic Plan (NSP) targeted a cumulative 4.3 million MMCs by the end of 2016 ^18^. Target setting, in terms of numbers and priority age groups, was informed by use of the projection tool Decision Makers' Program Planning Tool ^19^. Through the end of 2017, 3 459 935 MMCs had been reported. The 2017–2022 NSP targets a cumulative 3 million MMCs to be performed between 2016 and 2021 ^20^. PEPFAR, implementing MMC in 27 priority districts, aims to achieve 80% circumcision coverage in men 15–39 years by 2022 ^21^. The MMC program and its targets were underpinned by modeling studies that suggested that MMC scale-up is likely to be very cost-effective and could potentially have a large impact on South Africa’s HIV epidemic ^22, 23^.

As the MMC program strives to reach these 2022 targets in a context of limited resources and intervention options, it is timely to evaluate its actual health and economic impact and cost-effectiveness, based on actual program results to date. We applied three mathematical models of HIV in South Africa to examine the current and potential future impact of this program. This study was conducted jointly by three modeling groups, in cooperation with national program managers and stakeholders. We used three different models as implemented by three different modeling group to ensure that results were robust and not dependent on the specifications of a single model.

## Methods

### Structure of the three mathematical models

***Goals model.*** Avenir Health applied the deterministic Spectrum Goals model, previously applied in South Africa ^24– 26^ and other southern African countries (for example, ^27^). This compartmental, risk-structured model sits in the Spectrum platform, building on a demographic module that projects populations over time and models HIV epidemic spread between compartments of adults 15–49 years: low-risk adults who have one heterosexual partner; medium-risk adults with two or more partners in a year, high-risk adults who are female sex workers (FSW) and their clients, and men who have sex with men (MSM). Each group is characterized by numbers of partners, acts per partner per year, condom usage rate, and age at first sexual relationship, which can all be set to change over time, either as spontaneous social trends or in response to behavioral interventions; as well as proportion married (time-constant). The probability of HIV transmission is determined by type of contact, disease stage, MC status of male (uninfected) partners, condom usage, and ART status of the infected partner. Parameters determining the probability of HIV transmission are sampled from plausible ranges (established from literature) to optimally fit the historical epidemic. Adult HIV incidence and prevalence from Goals are fed into the linked Spectrum module AIM, which translates these into outputs such as numbers of people living with HIV (including pediatric infections), new infections, AIDS deaths, the need for ART, and prevention of mother-to-child transmission, by age group including those below 15 and above 49 years.

***EMOD Model.*** The Institute for Disease Modeling applied EMOD, an individual-based stochastic model with explicit age structure. EMOD **-**HIV version 2.20 describes HIV as transmitted through age-structured heterosexual coital acts between individuals in sexual partnerships as well as vertically through mother-to-child transmission. Four types of sexual partnerships (marital, informal, transitory, and commercial) are remembered over time and formed according to specifiable partner age patterns. Infectivity of HIV+ individuals depends on infection stage (acute, latent, AIDS), ART, STI co-infection and condom usage. Susceptibility is influenced by STI co-infection, condom usage, PrEP usage and for males, by MC on a per-coital-act basis. While most of the population is serially monogamous, medium-risk individuals can have concurrent relationships and high-risk female sex workers and their male clients have frequent short-term relationships in addition to potentially concurrent longer-term relationships. HIV testing occurs voluntarily, at antenatal visits, or once symptomatic. Individuals flow through a realistic representation of the ART continuum to access treatment and may discontinue and later resume care. Model fitting using Parallel Simulations Perturbation Optimisation ^28, 29^ was used to identify 250 unique parameter combinations that produce epidemic patterns consistent with historical data.

The EMOD South Africa model utilizes country-specific demographics (age-specific fertility and age/sex-specific non-AIDS mortality), ART (over time and by sex), and HIV prevalence estimates (by age and sex over the available survey years).

***Thembisa Model.*** The University of Cape Town applied the deterministic model Thembisa, a combined demographic and HIV model, which was developed for South Africa and which has previously been used to assess the relative impact of different HIV prevention and treatment strategies ^10, 30^. This compartmental, risk-structured and age-structured model divides the population into a number of demographic cohorts (defined by sex and individual age). Within each cohort, the population is sub-divided into further compartments, defined in terms of sexual behavior characteristics such as sexual experience, risk group (high or low risk), marital status and sexual preference (heterosexual or bisexual). The population is further stratified by HIV testing history (in the case of men) and circumcision status. HIV-positive individuals are divided into different CD4 groupings and are classified as undiagnosed, diagnosed but untreated, or treated. HIV transmission is modelled based on assumptions about coital frequencies, condom use and probabilities of HIV transmission per sex act, all of which depend on age, sex and relationship type. The model also allows for changes in condom use over time, as a result of HIV communication programmes. The model is fitted to age-specific HIV prevalence data from antenatal and household surveys, and is also calibrated to age-specific all-cause mortality data. A more detailed description of the model and the calibration procedure is provided elsewhere ^10^.

The rate at which men get circumcised is assumed to be composed of two parts: the ‘background’ rate of male circumcision that would be expected in the absence of any efforts to promote male circumcision as an HIV prevention strategy, and the rate of male circumcision due to MMC campaigns. In modelling the former, a cumulative Weibull distribution is used to represent the age-related changes in the prevalence of male circumcision prior to 2008. It is assumed that the prevalence of male circumcision at age *x* is determined by the function

$$p(x)=a+\left(b-a\right)\left(1-0.5^{{\left(x/m_{1}\right)}^{\phi }}\right),$$

where *a* is the proportion of males who are circumcised soon after birth, *b* is the maximum cumulative uptake of male circumcision in the absence of MMC promotion, *m*_1_ is the median age at circumcision in men who get circumcised after birth, and *ϕ* is the shape parameter that determines the concentration of the distribution of circumcision ages (post-birth) around the median. Since surveys usually report the median age at circumcision for all men (including those who are circumcised at the time of birth), it is useful to parameterize the model in terms of this overall median circumcision age, *m*_2_, noting that

m1=m2(ln⁡(b/(2(b−a)))ln⁡(0.5))−1ϕforb2>a.

**Figure 1.**
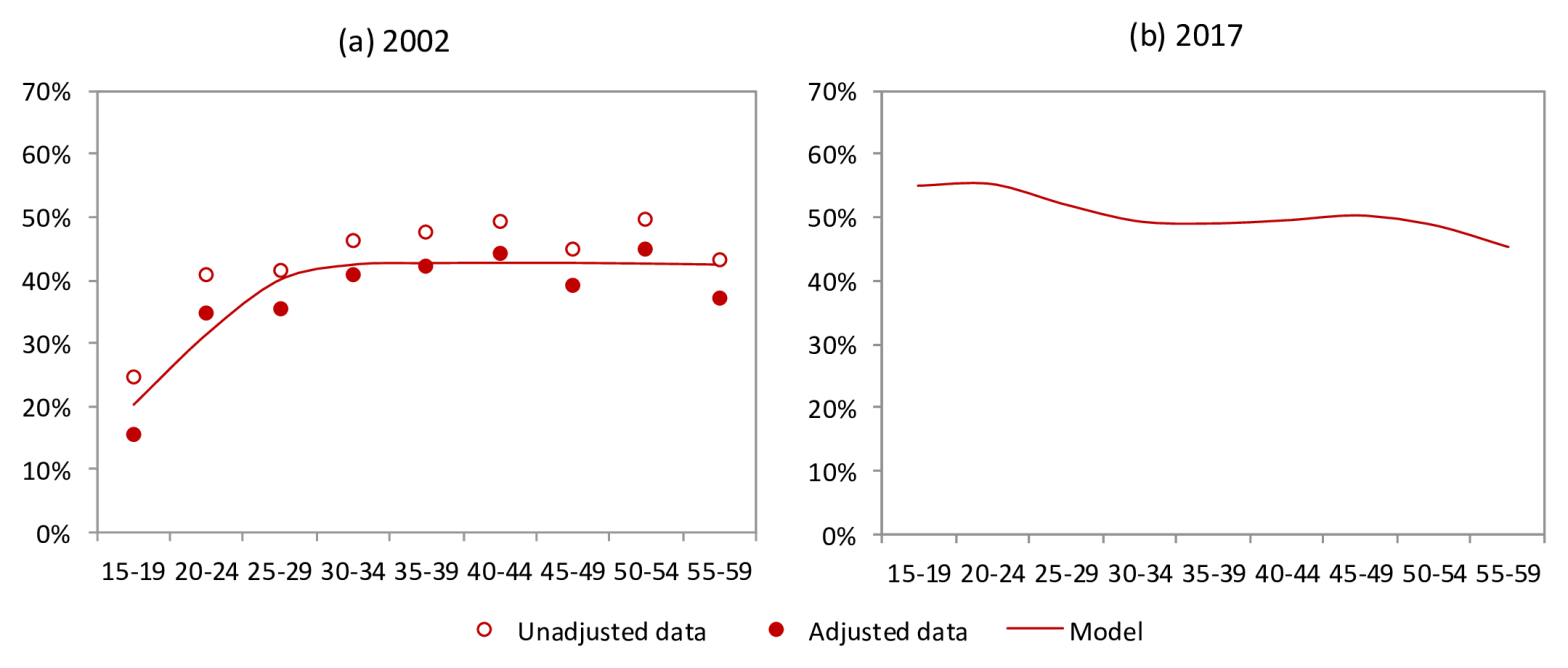
Fraction of men who are circumcised, by age: ( **a**) in 2002 just prior to MMC campaigns; ( **b**) in 2017. In ( **a**), Unadjusted data represent the average of the results from national surveys in 2002 and 2003 ^8, 31^; Adjusted estimates are calculated on the assumption that the sensitivity and specificity of self-reported male circumcision status (relative to true status) are 96.4% and 88.4% respectively ^32, 33^.

Parameters *a* and *b* are set at 0.105 and 0.42 respectively. The shape parameter *ϕ* is set at 4.5, and the median age at circumcision *m*_2_ is set at 18, the median age at circumcision reported by Africans in the 2002 HSRC and 2003 DHS surveys ^8, 31^. Most of these parameters have been set so that the model is consistent with reported rates of male circumcision by age in national surveys ^31^, after correcting the self-reported data to take into account known biases in the reporting of male circumcision ^32, 33^. This adjustment for mis-reporting of male circumcision excludes men who are partially circumcised, i.e. partially circumcised men are treated as uncircumcised. The two national surveys used in the parameterization were conducted in 2002 and 2003, and thus represent the situation prior to the promotion of male circumcision as an HIV prevention strategy. Figure 1 shows the model calibration for the proportion of men circumcised at baseline (in 2002, prior to start of the MMC program).

The annual probability that uncircumcised men aged *x* would get circumcised in the absence of MMC campaigns is calculated from the *p*( *x*) values defined previously using the equation

$$\psi (x)=\frac{p(x+1)-p(x)}{1-p(x)}.$$

Extending the model to include MMC in response to MMC promotion campaigns requires that we define the symbol *p* ^*^( *x*, *t*) as the proportion of men aged *x*, at time *t*, who are circumcised. Of those men who are uncircumcised at age *x* in year *t*, the proportion who intend to get traditionally circumcised (i.e., they would want to get circumcised even in the absence of MMC promotion campaigns) is calculated as

$$\frac{b-p(x)}{1-p^{*}(x,t)}\;,$$

and the proportion who do not intend to get traditionally circumcised is

$$\frac{1-p^{*}(x,t)\;-\left(b-p(x)\right)}{1-p^{*}(x,t)}\;.$$

The implicit assumption is that the men who intend to get traditionally circumcised would not accept MMC, i.e. the demand for traditional MC and the demand for MMC are mutually exclusive. This is different from the assumption made in the most recent previously-published version of Thembisa (Thembisa 4.1 ^10^), in which it was assumed that the demand for MMC was independent of the individual’s desire for traditional male circumcision. This change was made because the previous model produced estimates of circumcision coverage that appeared implausibly low relative to the levels reported in recent surveys (even after correcting for misreporting), and because data from the most recent HSRC household survey ^34^ suggest there has been no reduction in the prevalence of traditional male circumcision since the start of the MMC rollout (in contrast to what would be expected if some of the men who would previously have been traditionally circumcised instead chose MMC). With the revised assumption, the model yields a slightly higher estimate of male circumcision coverage, more consistent with recent survey data.

Men are assumed to undergo MMC only if they are HIV-negative, as HIV testing is conducted prior to most MMC operations ^35, 36^, and although men who are HIV-positive are not excluded from getting circumcised, there would be little incentive to undergo the procedure if they were already HIV-positive. The symbol *η*( *x*, *t*) is defined as the probability that HIV-negative men who are aged *x*, uncircumcised at the start of year *t*, and not intending to get traditionally circumcised, get medically circumcised through MMC campaigns. This is calculated as *η*( *x*, *t*) = *θ*( *t*) × *R*( *x*), where *θ*( *t*) is the maximum probability in year *t* and *R*( *x*) is the relative rate of MMC uptake in men aged *x*, compared to boys aged 10–14 years. The relative rates of MMC uptake in the 15–19, 20–24, 25–49 and 50+ age groups have been set to 0.59, 0.27, 0.14 and 0.012 respectively; these rates were chosen to ensure the model matched the age profile of MMC operations provided from PEPFAR-supported MMC programmes in South Africa. This results in the prevalence of male circumcision by age in 2017 shown in Figure 1b.

The *θ*( *t*) values are estimated from the reported number of MMC operations in year *t*, Λ( *t*). Mathematically,

$$\Lambda (t)=\sum_{x}N(x,t)\left(1-\frac{b-p(x)}{1-p*(x,t)}\right)\eta (x,t),$$

where *N*( *x*, *t*) is the number of uncircumcised, HIV-negative men who are aged *x* at the start of year *t*. From the above equation, it follows that

$$\theta (t)=\Lambda (t)/\sum_{x}N(x,t)\left(1-\frac{b-p(x)}{1-p^{*}(x,t)}\right)R(x).$$

Combining traditional and medical male circumcision, the net probability of male circumcision in a male aged *x* at the start of year *t* is

$$\psi (x,t)=\frac{p(x+1)-p(x)+(1-p^{*}(x,t)-(b-p(x)))\theta (t)R(x)}{1-p^{*}(x,t)}.$$

### Model calibrations to South Africa’s historical HIV epidemic

Each of the three models was independently calibrated to historical data on South Africa's HIV epidemic. All three models were fitted to the national epidemic; additionally as a sensitivity analysis, the Thembisa model was fitted for each of South Africa’s nine provinces, and alternative national estimates were obtained by aggregating across the provinces.

Key modeling assumptions were standardized across all models. Fertility and mortality were set according to census data and demographic estimates (for Goals and EMOD, from the 2017 UN World Population Prospect (WPP) ^37^; for Thembisa from South Africa national demographic estimates ^10^).

Key epidemic data for model calibration were sero-prevalence estimates in adult men and women from national surveys conducted in 2002, 2005, 2008, 2012 and 2017 conducted by South Africa’s Human Sciences Research Council (HSRC) ^5– 7, 9^, as well as time trends in prevalence from national antenatal clinic surveys ^38^.

**Figure 2.**
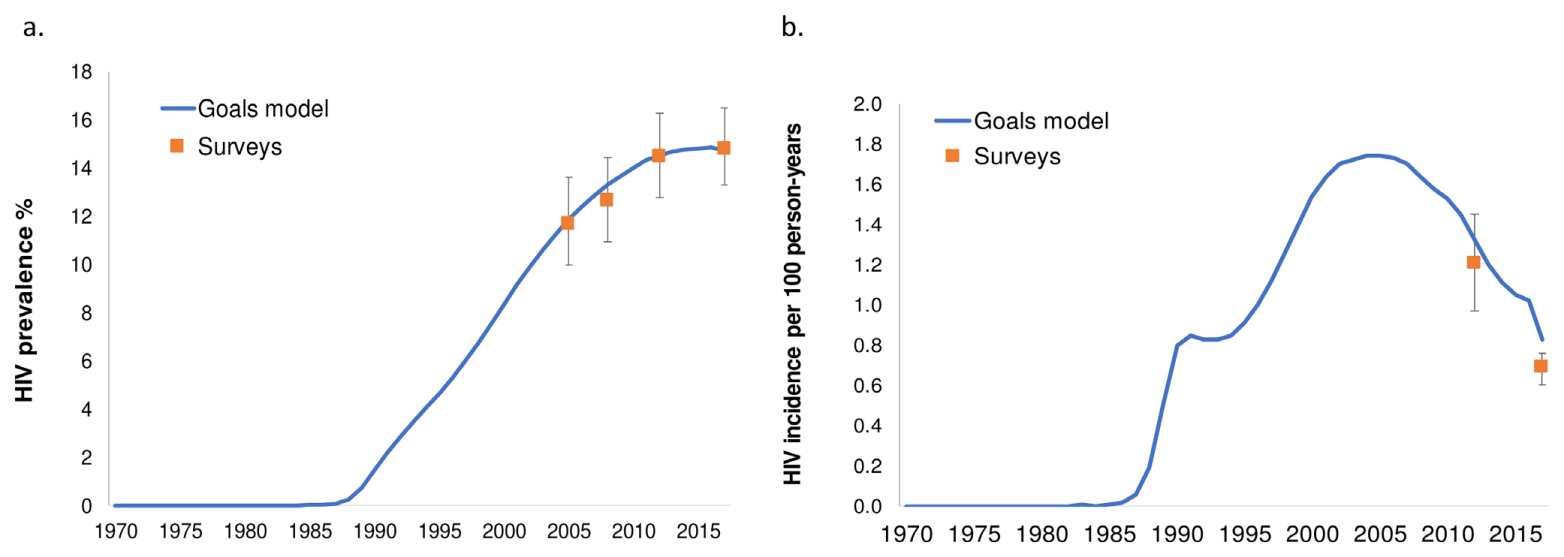
Goals model calibration to national HSRC survey data, for ( **a**) HIV prevalence, and ( **b**) HIV incidence in men 15–49 years. model outputs (blue lines = average across simulations; grey lines = individual simulations) compared to data from HSRC surveys (black dots).

**Figure 3.**
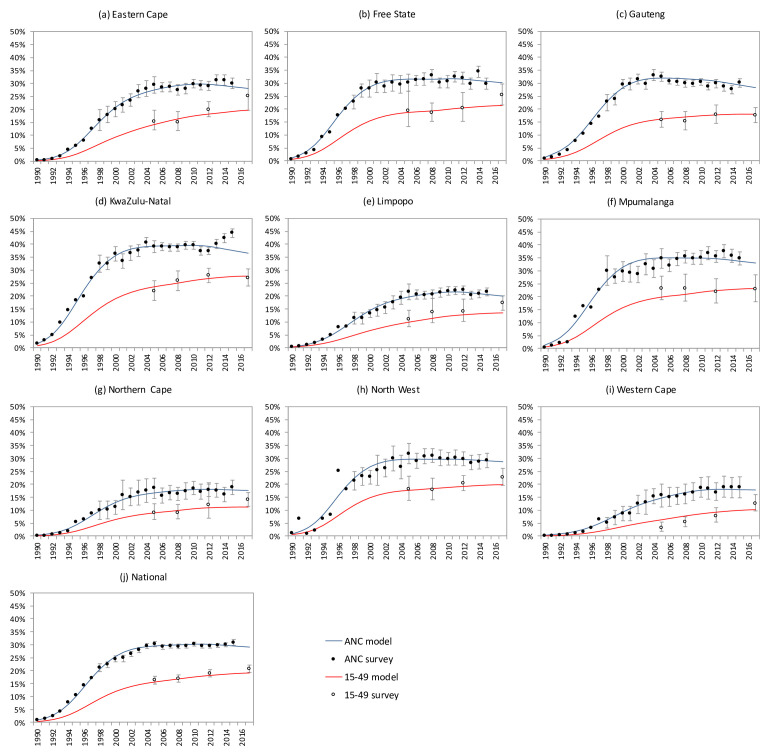
Thembisa model calibrations: provincial-level HIV prevalence produced by the calibrated model in the 15–49 population (red) and pregnant women (blue) in comparison with time trends from antenatal clinic surveillance (closed circles), and prevalence data from the Human Sciences Research Council (HSRC) surveys (open circles).

**Figure 4.**
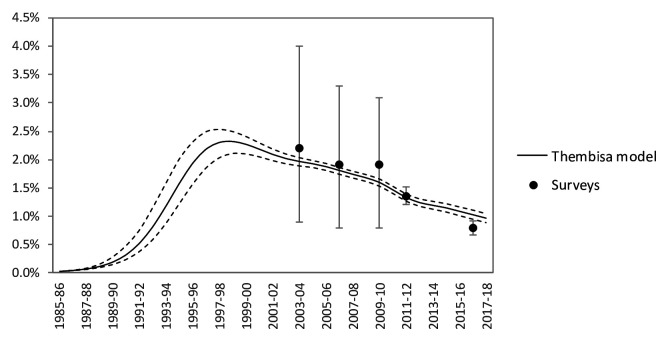
HIV incidence rates per annum in the 15–49 population. Solid line represent the average HIV incidence rate estimated by Thembisa; dashed lines represent 95% confidence intervals around the national estimates. Dots represent Human Sciences Research Council (HSRC) survey-based estimates of HIV incidence; the first 3 estimates are based on a synthetic cohort estimation approach, while the most recent 2 estimates are based on a multi-assay algorithm for detecting recent HIV infection ^47^.

**Figure 5.**
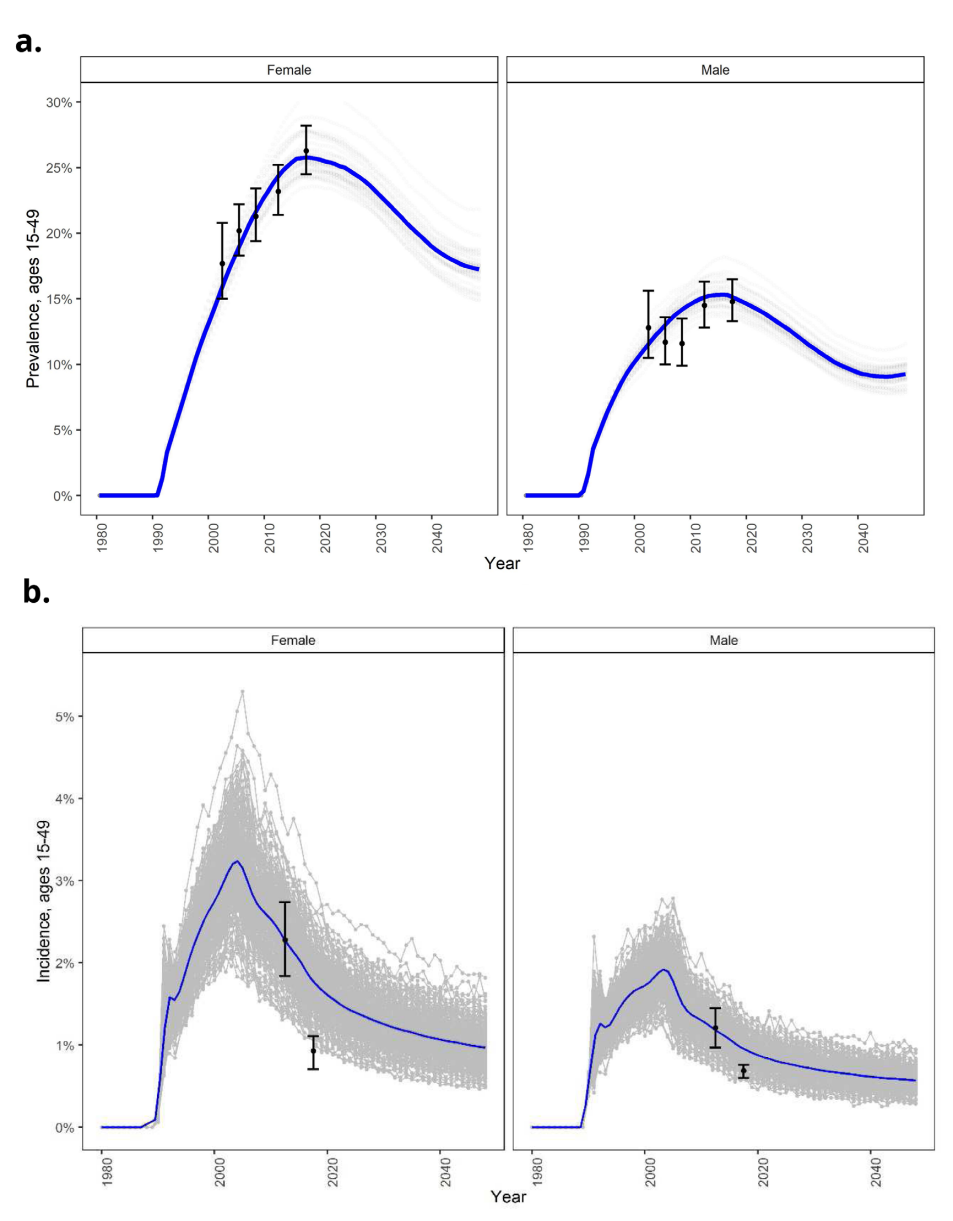
EMOD model calibration: ( **a**) National HIV prevalence in women and men 15–49 years over time; ( **b**) National HIV incidence in women and men 15–49 years over time.

Historical patterns of risk behavior and condom usage, which determine historical HIV transmission in the models, were informed by data from the HSRC surveys, national Demographic and Health Surveys (DHS) in 1998 ^39^, 2003 ^8^ and 2016 ^40^, national HIV communication surveys conducted in 2009 and 2012 ^41, 42^, and epidemiological, behavioral, and intervention studies in South Africa. HIV transmission probabilities and disease progression rates were fitted, within plausible ranges established by longitudinal studies in the South and East African region, to produce the best fit in terms of HIV prevalence by gender nation-wide (all models), by province (Goals and Thembisa) and by age (Thembisa and EMOD). Numbers of adult men, adult women, and (in Goals and Thembisa) children under 15 years living with HIV who were on ART over 2000–2016 were estimated from national HIV program statistics. The resulting calibrated models are shown in Figure 2 (Goals), Figure 3, and Figure 4 (Thembisa) and Figure 5 (EMOD).

The effectiveness of MMC was set conservatively, as a direct reduction in female-to-male transmission probability per sex act of 60% ^11– 16^. All models also included onward transmission capturing the indirect effects on women as a result of reduced male prevalence and the subsequent indirect effects on men. We used a cost of US$ 132 per procedure, for surgical MMC, which was the only or predominant method used up to 2015, based on a national ingredients-based costing study in 33 public facilities including urban and rural dedicated MMC delivery points, integrated clinics, and outreach facilities ^43^. This covered direct and indirect cost including community mobilization and demand creation ^43^. The unit cost of (first-line) ART was set at US$ 342 per adult per year, including drugs, laboratory costs, and service delivery, estimated from the South Africa government’s perspective ^44, 45^.

### MMC delivery and coverage, 2010–2017

The prevalence of traditional male circumcision was determined from self-reported male circumcision rates in national HIV surveys conducted prior to the start of the MMC program and surveys since 2010 were used to indicate current prevalence of circumcision from all sources. We used data from seven national surveys: South Africa’s Human Sciences Research Council (HSRC) surveys of 2002, 2008, 2012 and 2017 ^5– 7^, a national Demographic and Health Survey (DHS) in 2003 ^8^, and the 2009 and 2012 national HIV communication surveys ^41, 42^.

Data on MMCs performed between 2013 and 2017 in the public sector by district are contained in the District Health Information System (DHIS). Before this, data were collected at provincial level. Under-reporting was anticipated from MMCs conducted at community level through outreach or campaign events or mobile clinics if they were not reported to the local clinic. Another source of potential underreporting was from General Practitioners contracted by the Department of Health and not registered on the DHIS. Numbers of MMCs performed by province were taken from the national DHIS. These include MMCs performed in the public sector (Extended Data file 1, section A ^46^); we added private-sector MMCs as an average of 2.2% of MMCs in any year, based on data provided by medical schemes, and added data from the PEPFAR database (Datim) for the large MMC campaign conducted in the winter of 2017–2018, which had not been fully captured in the DHIS.

The age distribution of men receiving MMC was obtained from the Datim database, since DHIS lacked age stratification. Based on Datim/PEPFAR, 43% of MMCs for the 10+ years age group were in boys 10–14 years, 23.7% in 15–19 years, 12.3% in men 20–24 years, 20.1% in the 25–49 year age group and 0.9% in men above 49 years. In the Thembisa and EMOD models, these MMCs were distributed directly to male individuals of the appropriate age. For the Goals model (without age structure), MMC numbers were translated into proportions of 15–49-year-old men circumcised, by applying an age-structured population cohort calculation.

All three models assumed (optimistically) that there is no replacement of traditional MCs by MMCs (i.e. the proportion of men who get traditionally circumcised remains constant over time). EMOD did not age-stratify the rate of traditional male circumcision; rather it assumed 42% of adult men in all age groups to be circumcised, as a way of assuming that traditional circumcision always took place prior to sexual debut. In contrast, MMCs could be conducted either before or after sexual debut. The age distribution of MMCs in EMOD and Thembisa model followed the age-stratified estimates provided by the Ministry of Health. For the 2018–2030 scale-up, all three models applied the same age structure as in the 2009–2017 program data.

For MMC, South Africa’s MMC policy prioritizes HIV-uninfected men. MMC candidates diagnosed with HIV at enrolment are referred for enrolment into ART, evaluated for clinical fitness for MMC, and offered MMC after enrolling into ART. Among program-recorded MMCs, 98% were in HIV-uninfected men. Thembisa and EMOD assumed that all MMCs were performed in HIV-negative men, while the Goals model assumed equal uptake of MMCs by HIV-positive and HIV-negative men (details in Extended Data File 1, section B ^46^).

### MMC scale-up scenarios

We modeled three scenarios to estimate the effects of the MMC program.

- No MMC. This scenario assumes that the medical circumcision program was never implemented. The prevalence of male circumcision remains constant at the 2008 level through 2030.
- MMC program stopped after 2017. This scenario includes the actual increases in circumcision coverage from 2010 to 2017 and then assumes that the program stopped, so that circumcision coverage changes after 2017 only due to the ageing of the population (see underlying data ^46^).
- 2021 program targets achieved. In this scenario, the annual MMC program targets for 2017–2021 are met and additional circumcision are performed after 2021 in order to maintain the prevalence achieved in 2021.

For future coverage of ART and non-MMC prevention interventions, all three scenarios assume a 'status quo' setting, as described in detail for the Thembisa model ^10^. For ART, we assumed a gradual ongoing increase in coverage, from 56% in 2017 to around 67% by 2021 and to around 72% by 2025, based on Thembisa projections assuming rates of ART initiation after diagnosis and rates of ART interruption in future are similar to those estimated in recent years. For condom use, we assumed no changes after 2017, and we assumed no significant roll-out of Pre-Exposure Prophylaxis. Also risk behaviours (commercial and casual sex and concurrent partnerships) were assumed to remain unchanged from the levels in 2017.

The three models assumed a considerable effect of ART on reducing HIV infectivity and transmission, via viral load suppression (VLS): 75% reduced per-act transmission in Goals, between 84–91% in Thembisa (based on 78–86% VLS, according to program VLS monitoring data over 2005–2015 ^10^), and 92% reduced per-act transmission in EMOD ^48^.

The number of infections averted and the additional costs of the MMC program were calculated as the difference between the two scale-up scenarios and the ‘No MMC’ scenario. We compared result for the historical period (2009–2017) and the future (2017 through 2030). Ranges for our results are based on difference among the three models (see underlying data ^46^).

### Statistical analysis

The modeling analyses described here were conducted using the Goals module of the Spectrum software version 5.73, the Thembisa model version 4.1, and the EMOD HIV software version 2.20.

## Results

### MMC scale-up

The Goals model estimated the coverage of traditional MC to have been 43% of men aged 15–49 years in 2008. Thembisa estimated this to have been 36% and EMOD around 37% in 2008 and 38% in 2017. Both Thembisa and EMOD assume a net over-reporting of MC, i.e. that many of the men who report being circumcised are either not circumcised or only partially circumcised ^32, 33^.

**Figure 6.**
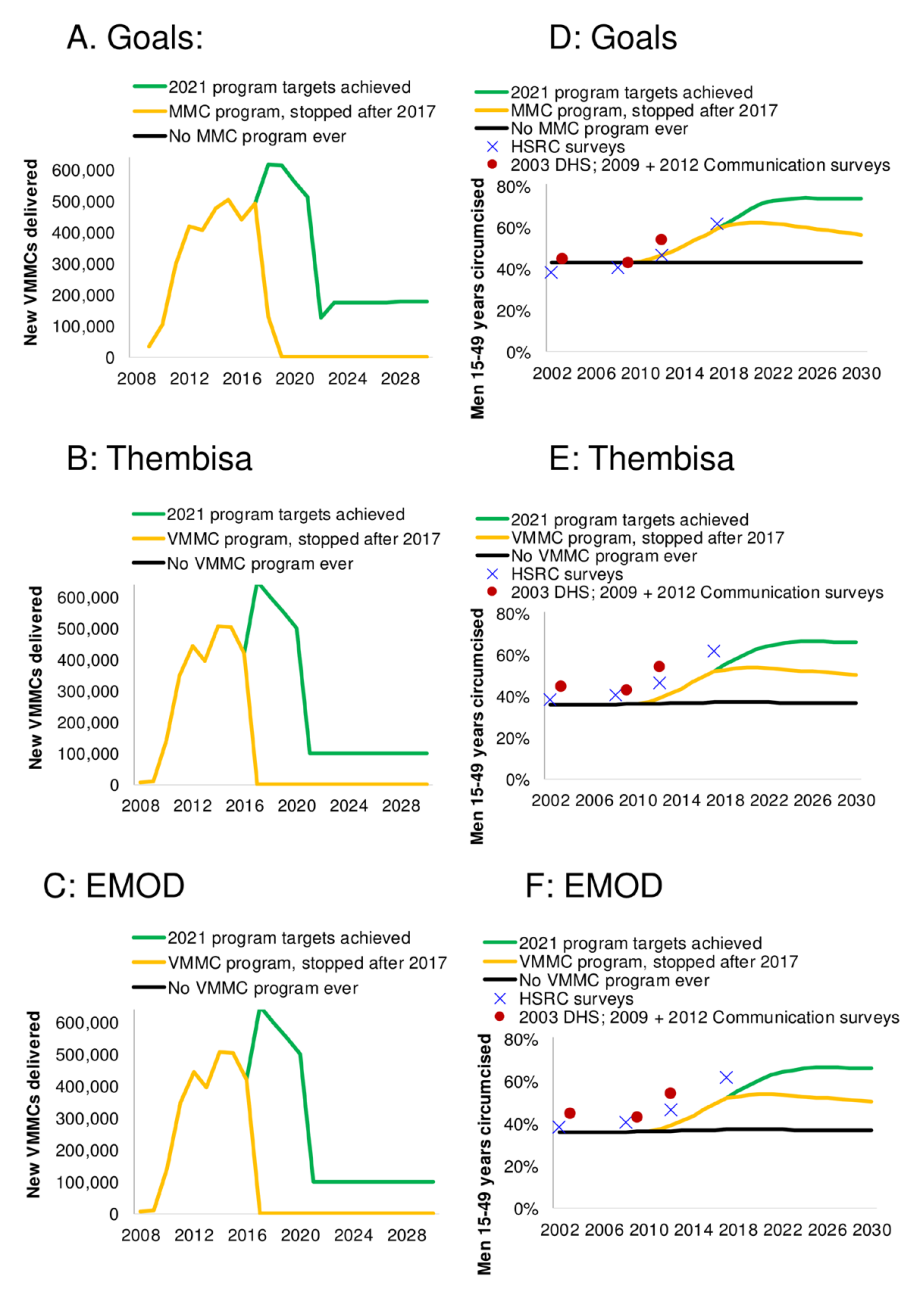
Modeled circumcisions: **A**, **B** and **C**: Number of new Medical Male Circumcisions (MMCs) occurring each year, and ( **D**, **E** and **F**) the resulting percentage of men ages 15–49 who are circumcised, by scenario in ( **A** and **D**) Goals, ( **B** and **E**) Thembisa and ( **C** and **F**) EMOD model. The 2009–2017 new MMCs are from program data ^49^; the 2009 baseline circumcision coverage was the modelers’ estimate based on 2002, 2008, 2012 and 2017 Human Sciences Research Council (HSRC) surveys ^3, 5– 7^, 2003 DHS ^8^, and the 2009 and 2012 national HIV communication surveys ^41, 42^.

The output from all three models is consistent with program data and targets ^38^ as shown in Figure 6, e.g. 60% in 2017 ( Figure 6d, e and f; details in underlying data ^46^).

In ‘No MMC’ scenario, coverage in the 15–49 year old cohort falls gradually back to the baseline level of 43% as uncircumcised 14-year-old boys age into the cohort and circumcised 49-year-old men age out ( Figure 6d, e and f).

If the 2021 targets are achieved and maintained, then annual number of MMCs drop sharply after 2021, once the ‘catch-up’ phase is complete. During the subsequent 'maintenance' phase, circumcision prevalence is maintained by circumcising only 15 year-old boys. The proportion circumcised among men 15–49 years continues to slightly increase (until 2027 in Thembisa, or until 2035 in Goals), as the MMCs conducted up to 2021 in boys aged under 15 years add to the cumulative coverage among 15–49-year-old men.

### Impact of the MMC program, 2009–2017

**Figure 7.**
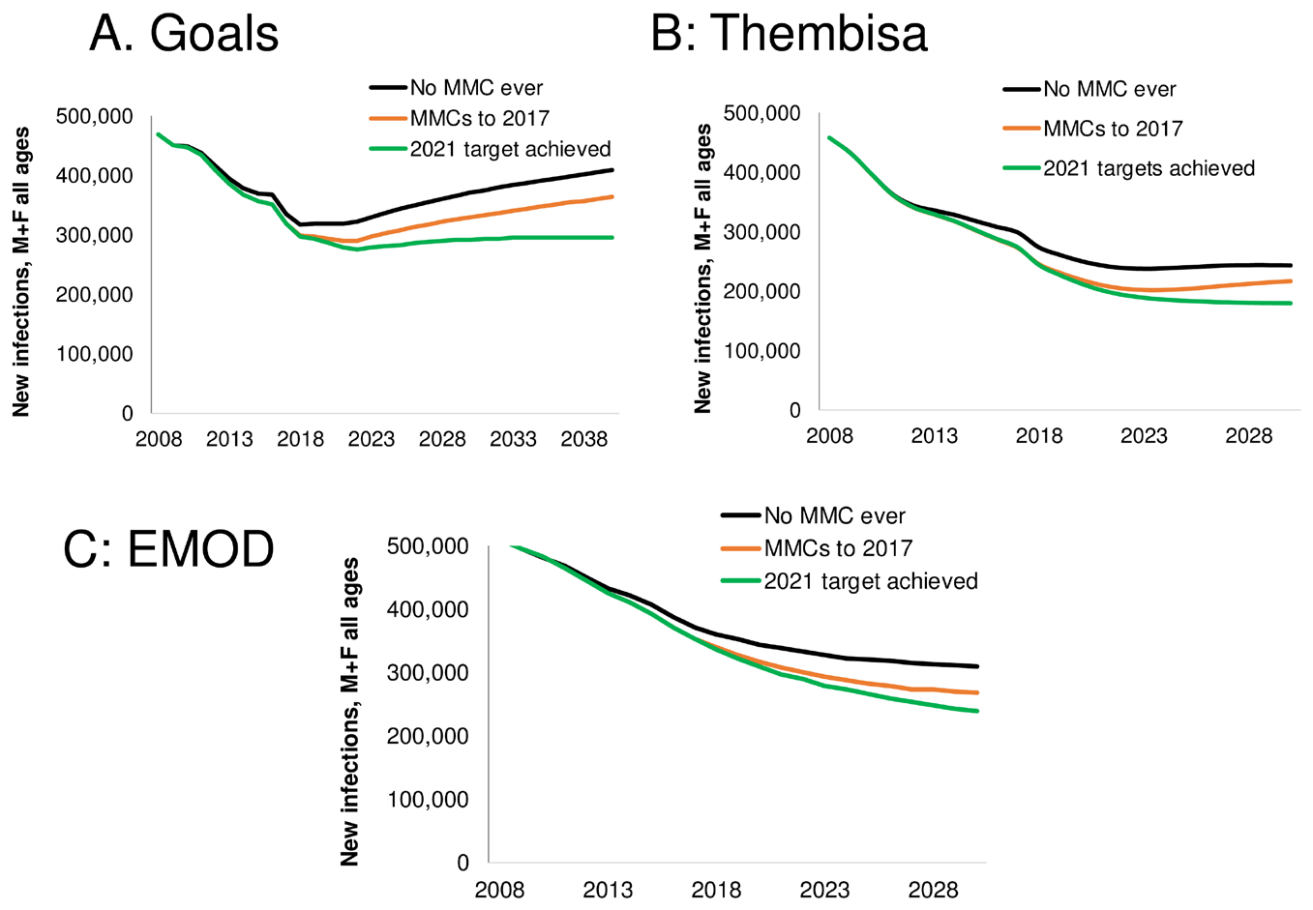
Number of new HIV infections each year over 2009–2040 by scenario, produced by the ( **A**) Goals, ( **B**) Thembisa and ( **C**) EMOD models. The scenarios shown are: the counterfactual of no Medical Male Circumcision (MMC) ever (black), the MMC program ends after 2017 (orange/red), the program targets through 2021 are met and maintained in a 'status quo' context (green). Infections are among all ages.

Across the three models, the MMC program has averted an estimated 71,000–83,000 HIV infections over 2009–2017 ( Figure 7 and Table 1). This implies 33–46 circumcisions or a cost of $4,400-$6,000 to avert one new infection ( Table 1).

**Table 1.** Projected impact, costs and savings from the MMC program in a 'status quo' background context, relative to the counterfactual scenario of no MMC program ever.

Scenario	Outcome	Goals model	Thembisa model *	EMOD
Results over 2009–;2017	Number of MMCs performed	3,175,986	2,763,444	3,233,009
	Number of HIV infections averted	73,663 (2%)	82,985 (2%)	70,731 (2%)
	MMCs per infection averted	43	33	46
	Cost per infection averted	$ 5,691	$ 4,396	$ 6,034
Results 2009–;2030; scenario Program ends after 2017	Number of MMCs performed	3,175,986	2,763,444	3,233,009
	Number of HIV infections averted	499,048 (6%)	496,837 (7%)	517,851 (6%)
	MMCs per infection averted	6.6	5.6	6.2
	Cost per infection averted	$ 874	$ 734	$ 824
Results 2009–;2030; scenario Program 2021 targets met and maintained	Number of MMCs performed	7,013,196	6,063,445	6,668,187
	Number of HIV infections averted	760,227 (9%)	747,441 (11%)	723,926 (8%)
	MMCs per infection averted	9.2	8.1	9.2
	Cost per infection averted	$1,218	$1,071	$ 1,218
	Savings in ART costs, 2018–;2030	$ 1,159 million	$ 678 million	$ 601 million

The percentages in parentheses represent the proportion of new HIV infections averted relative to the number of new HIV infections in the counterfactual scenario. Outcomes are for all ages (0–99 years). MMC = Medical Male Circumcision * Thembisa numbers in the first row relate to MMCs performed over the period from 1 April 2009 to 31 March 2017.

### Long-term impact of MMC

Next we evaluated the impact of actual recent, and targeted new MMCs over the longer timeframe of 2009–2030 ( Table 1). The MMCs performed up to 2017 will avert 497,000–518,000 new infections (6–7% of all new infections) over 2009–2030, even if no further MMCs are performed.

Achieving the MMC targets by 2021, with maintenance thereafter, will avert 724,000–760,000 infections (8–11% of all new infections) over 2009–2030. MMC is an efficient intervention, requiring only 8–9 circumcisions per infection averted.

Scaling-up to reach the 2021 targets and maintain that higher circumcision prevalence will prevent new infections compared to the ‘No MMC’ scenario, but will also require additional expenditures. Over 2009–2030, the cost to avert one HIV infection with MMC will be $1,070–1,220.

**Figure 8.**
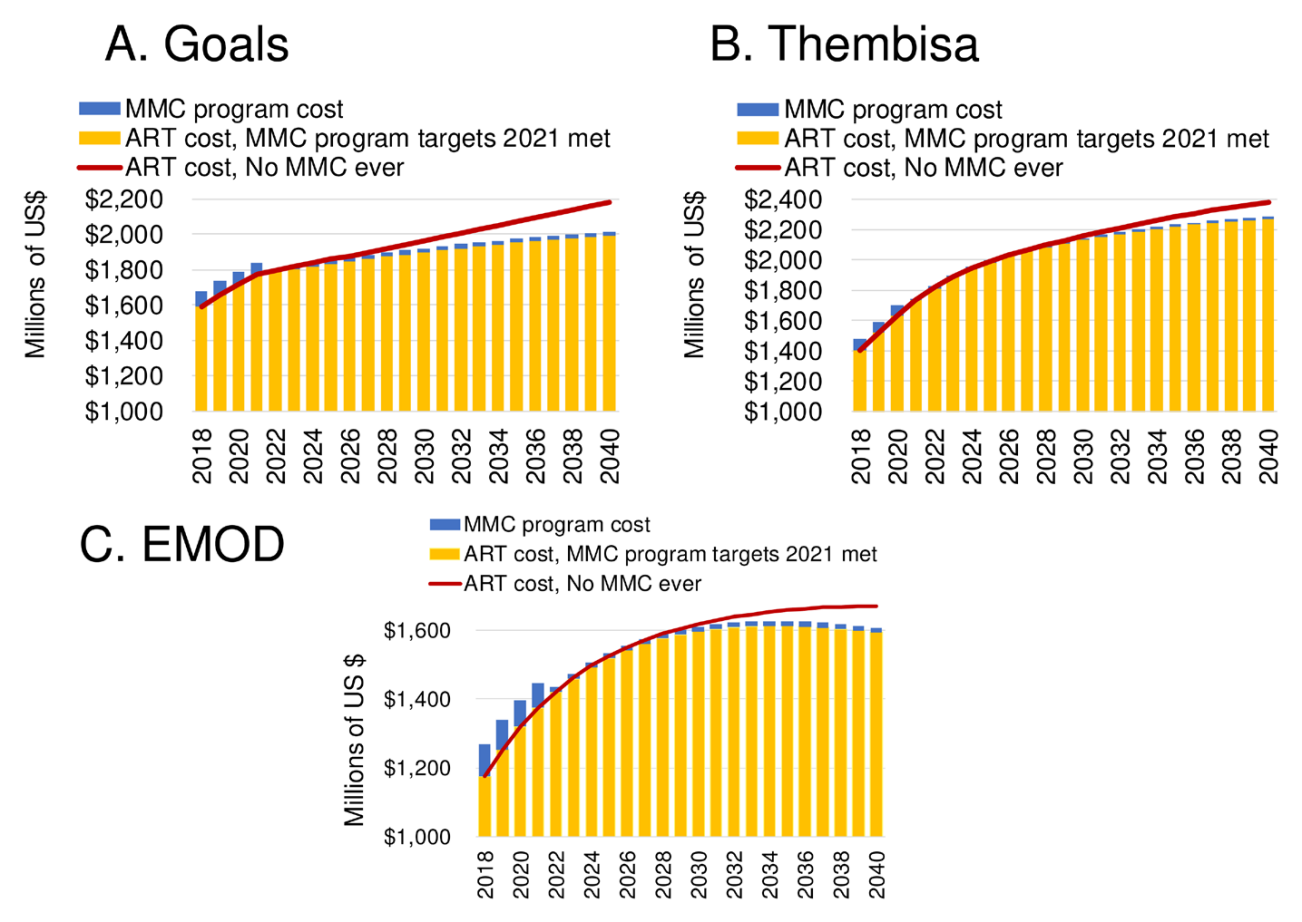
Costs of MMC and ART in the scenario in which the program targets for 2021 are achieved in a 'status quo' setting (without Fast Track), compared to the counterfactual scenario of no Medical Male Circumcisions (MMCs) ever, from ( **A**) Goals, ( **B**) Thembisa, and ( **C**) EMOD model. Shown are the cost of Medical Male Circumcision (MMC) if the program targets are met and maintained (blue bars), the cost of ART if the program targets are met and maintained (orange bars), and the cost of ART in the counterfactual scenario with no MMC ever (red lines).

Infections averted by MMC reduce the future ART treatment need and cost, by $ 602–1,159 million over 2018–2030 ( Table 1). Once the 2021 targets are achieved, the annual cost of maintaining circumcision coverage will drop by 70–80% while the savings from treatment averted will continue to grow. A ‘break-even’ point occurs when the cumulative savings from treatment averted exceed the cumulative costs of the MMC program. This occurs in 2034 in Goals, in 2036 in Thembisa model, and in 2038 in EMOD ( Figure 8).

### Sensitivity analyses

The results of our sensitivity analyses are shown in Table 2. The main analysis did not discount future costs or infections averted. As an alternative we applied a 3% annual discount to future impacts and costs, using 2017 as the reference year. Over 2009–2030, discounting increases the cost of MMC per infection averted from $1,070–1,218 to $1,244–1,400, because health impacts lag costs.

**Table 2.** Sensitivity analyses. Impact and cost-effectiveness of South Africa’s MMC program, under alternative assumptions for key parameters whose values are uncertain.

Model variant	Infections averted			Cost ^a^ per infection averted			Break-even point *			Savings from ART averted, millions of US$ *		
	Goals	Thembisa	EMOD	Goals	Thembisa	EMOD	Goals	Thembisa	EMOD	Goals	Thembisa	EMOD
*Default*	*760,227*	*747,441*	*723,926*	*$ 1,218*	*$ 1,071*	*$ 1,218*	*2034*	*2036*	*2039*	*1,159*	*678*	*602*
HIV infections averted and costs discounted at 3% per year after 2017	610,103	609,507	528,132	$ 1,402	$ 1,244	$ 1,416						
Add MMC protective efficacy reducing M-to-F transmission by 46%	1,373,565	NA	NA	$ 674	NA	NA						
ART and non-MMC prevention interventions scaled up according to ambitious UNAIDS global Fast Track targets (Extended Data File 1, section C), instead of status quo	47,638	NA	105,141	$ 10,633	NA	$ 4,313						
Epidemics and MMC scale-up modelled for each of 9 provinces separately, instead of as national aggregate.	NA	743,717	NA	NA	$ 1,046	NA						
MMC unit cost: $ 198 instead of default $ 132	As default			$ 1,827	$ 1,608	$ 1,826	2037	2039	2043	As default		
MMC unit cost: $ 66 instead of default $ 132	As default			$ 609	$ 536	$ 609	2029	2032	2034	As default		
ART unit cost: linear decline from $ 342 in 2017 to $ 293 from 2026	As default			As default			2035	2037	2041	1,018	590	527
ART unit cost: $ 400 over 2018– 2030, instead of default $342	As default			As default			2032	2035	2038	1,356	792	704

The results shown are for the scenario in which the program targets for 2021 are achieved in a 'Status quo' context, with infections averted, cost per infection averted and savings from ART averted over 2009–2030. ^a^ Cost is direct MMC program cost, not considering savings from ART averted ^d^ Default assumptions: No discounting, national aggregate models, MMC protective efficacy on female-to-male transmission 60% per sex act, no direct effect of MMC on male-to-female transmission. NA = Not evaluated. MMC = Medical Male Circumcision * The break-even point is the year by which the cumulative savings from ART averted become larger than the cumulative MMC investment cost, indicating that MMC is cost-saving. For scenarios explored that did not alter the number of infections averted by MMC, the break-even year and the savings from antiretroviral therapy (ART) averted were selected as key outcome of the sensitivity analysis

We assumed that MMC had direct effects only on female-to-male transmission. An analysis by Hallett *et al.* ^39^ indicated that there might be direct effects on male-to-female transmission as well. When we add a 46% protection effect against male-to-female transmission, the impact in terms of infections averted in the Goals model is 1.8-fold larger, and cost-effectiveness 1.8-fold better, than in the default results ( Table 2).

If instead of remaining constant, the coverage of ART and other interventions continues to increase, then incidence will decline and MMC will be less cost-effective. Specifically, if coverage of other interventions reached UNAIDS Fast-Track targets for 2030 ^50^ (Extended Data File 1, section C ^46^) then the number of MMCs needed to avert one infection and the cost to avert one infection are both higher ( Table 2). Even in this case the MMC program would avert 105,141 new infections (1.2%) in EMOD model or 45,638 (0.6%) in Goals model over 2008–2030. In the year 2030, new infections under Fast Track would be 11% (EMOD) or 7% (Goals) higher without MMC scale-up than with MMC scale-up.

The relation between the number of new infections in the No-MMC scenario and the impact and cost-effectiveness of MMC was assessed by examining correlations across individual simulations in the Thembisa and EMOD models, where all parameters were allowed to vary simultaneously within their respective uncertainty ranges (Extended Data file 1, section D ^46^). There was a positive correlation between the number of HIV infections at baseline and the impact of MMC, and a negative correlation between baseline HIV infections and the cost per infection averted by MMC.

According to another similar correlation analysis, the impact and cost-effectiveness of MMC were relatively insensitive to the effectiveness assumed for ART in reducing infectiousness (Pearson correlation coefficient 0.13, using the Thembisa model; Extended Data file 1, section D ^46^).

The main analyses assumed a constant ART unit cost of US $342 per person per year. But costs of ART are declining due to lower drug prices (particularly with the switch to dolutegravir) and differentiated service delivery. If we assume a linear decline from $342 in 2017 to $293 ^45^ by 2026, then the savings from averted ART costs due to MMC over 2018–2030 are $527–1,018 million, rather than the default $ 602–1,159 million; and the break-even point would be postponed by 1 or 2 years in each model. Conversely, if we assumed a higher ART unit cost of $400 (throughout 2009–2030) – which could reflect the higher average unit cost if we consider patients on more expensive second-line ARVs – then the savings from averted ART costs due to MMC over 2018–2030 would be $704–1356 million, and the break-even point would be 1 or 2 years earlier across all models.

Varying MMC unit cost to 50% more or less than the default cost assumption based on country data, changed the cost per infection averted by MMC linearly ( Table 2); the break-even point would be postponed by 3–4 years with a 50% higher MMC unit cost, or fall 4–5 years earlier in case of a 50% lower MMC unit cost ( Table 2).

**Figure 9.**
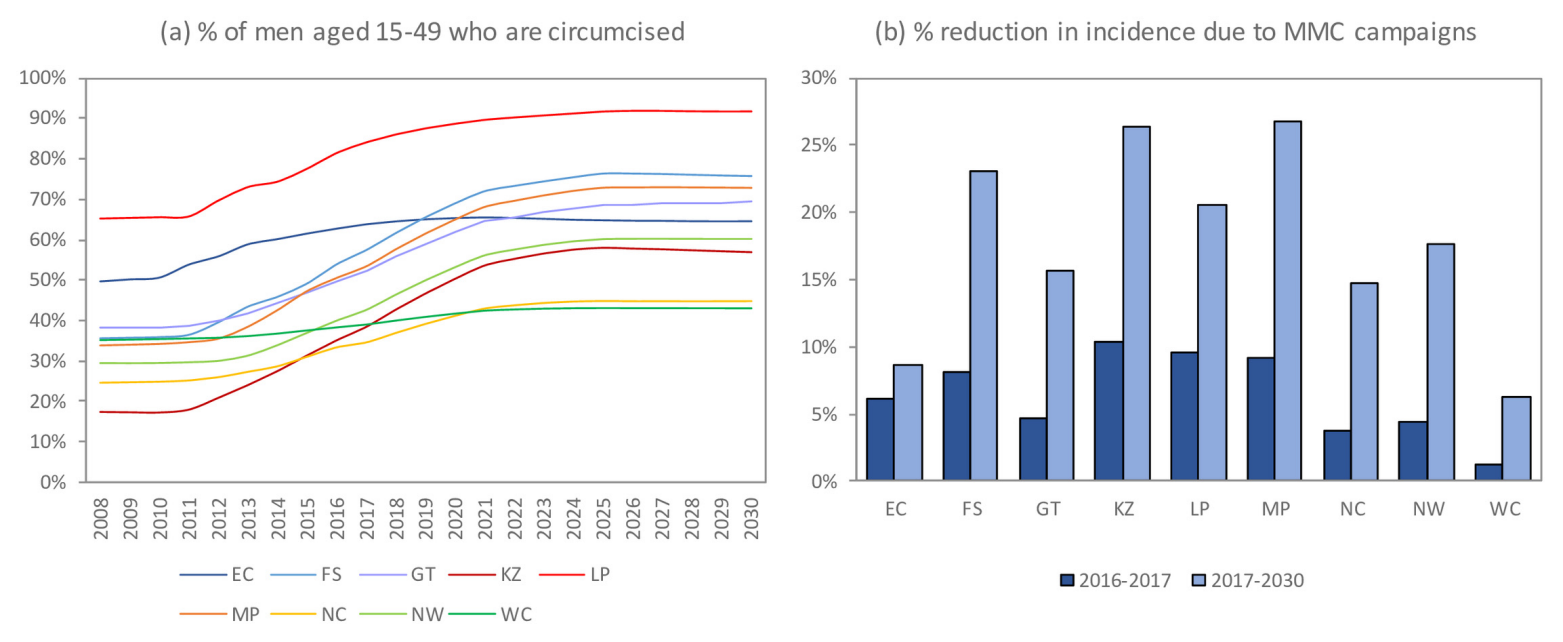
Provincial differences in MMC rollout and impact on HIV incidence. In the Thembisa provincial model for Limpopo, age-specific limits on the uptake of Medical Male Circumcision (MMC) were exceeded, with the result that the modelled number of MMC operations exceeded that reported by the program in some years (2015–2021). EC = Eastern Cape; FS = Free State; GT = Gauteng; KZ = KwaZulu-Natal; LP = Limpopo; MP = Mpumalanga; NC = Northern Cape; NW = North West; WC = Western Cape.

Finally, when MMC scale-up and impacts were modelled for each of South Africa’s 9 provinces and the results aggregated, in the Thembisa model ( Table 2 and Figure 9), the aggregate national results were similar to those of the (default) national model.

### Impact by province

Results differed substantially by province. Over the 2016–17 period, the Thembisa model estimated that the reduction in new HIV infections as a result of the MMC programme varied between 1% in the Western Cape province and 10% in the KwaZulu-Natal and Limpopo provinces, reflecting substantial inter-provincial differences in the extent of MMC scale-up ( Figure 9b). Assuming that the distribution of MMC operations occurring in each province will remain roughly unchanged up to 2021, and assuming the male circumcision coverage in each province remains roughly stable after 2021, it is anticipated that the Western Cape and Northern Cape provinces will have the lowest rates of male circumcision in future, while the KwaZulu-Natal, Free State, Mpumalanga and Limpopo provinces will have achieved the most substantial growth in male circumcision coverage ( Figure 9a). Over the period from 2017 to 2030, the reduction in total new HIV infections due to MMC promotion is expected to vary between 6–8% in the Eastern Cape and Western Cape provinces and 23–27% in the Free State, KwaZulu-Natal and Mpumalanga provinces ( Figure 9b).

## Discussion

Our results indicate that the South African MMC program has already had an impact on reducing new HIV infections and that impact will grow substantially in the future as the full benefits of the past program are realized. Achieving the targets of the MMC strategy will increase the benefits even more. Since MMC is a one-time intervention, the cost per infection averted is low compared to most other HIV prevention interventions. Other work ^27^ has suggested that MMC also has a comparatively low cost per DALY averted in comparison to key global non-HIV public health/infectious disease interventions ^51– 54^. We show that investments in MMC will be more than offset by savings in terms of reduced expenditures for future treatment. MMC remains beneficial even if other prevention targets, as specified in Fast-Track, are reached.

Overall, our combined results are in line with earlier evaluations of MMC cost-effectiveness in other sub-Saharan African settings; for example, MMC’s cost per infection averted (which ignores future savings from ART averted) was estimated at between $131 and $3,160 across three regions in Mozambique ^27^ and at a median of $4,400 across 14 priority countries in eastern and southern Africa ^55^.

A similar modeling study in Zimbabwe ^47^ that also used the Goals and EMOD models (but not Thembisa) plus a model from Imperial College found that cost per infection averted was good but at $2,100–3,250 per infection averted was twice as expensive as in South Africa ^56^ and the potential savings were less than in South Africa. The better results in South Africa are likely due to higher incidence and higher cost per person-year of ART.

Cost per infection averted was estimated to be even better in a study that modelled hypothetical MMC scale-up starting in 2006 for South Africa ($181–551) ^57^, reflecting the higher baseline incidence over this early evaluation period.

Our analysis and other modeling studies ^19, 27, 55, 57, 58^ show the benefits of direct protection for men and indirect protection for women. The benefits are even larger if there is also a direct effect on male-to-female transmission. Other benefits of MMC include reductions in sexually transmitted bacterial and viral infections such as herpes simplex virus type 2, syphilis and human papillomavirus ^59, 60^, and associated penile and cervical cancers ^61, 62^ in the circumcised men and their female partners , and the opportunity through pre-MMC HIV testing to link HIV-positive men into care. The external benefits not considered here furthermore include capacity building of providers including in broader HIV prevention counselling, and linkage of MMC clients to other men’s health programs.

### Limitations

The validity and precision of model-based results depend on the validity of input data and assumptions including model structures. The similarities in our results across the models indicates that these results are robust across different model structures (Extended Data file, section B ^46^). However, certain variations in results across the three models indicates some real remaining uncertainty, stemming from both ambiguities with input data and model-related uncertainties.

One uncertainty remains the current level of HIV incidence, which drives absolute numbers of infections averted. The 2012 HSRC survey measured HIV incidence among adults (ages 15–49), using a multi-assay algorithm that included Limiting-Antigen Avidity Enzyme Immune Assay, at 1.21 (0.97–1.45) and 2.28 (1.84–2.74) per 100 male and female person-years, respectively ^6^. All three models predicted somewhat lower incidence rates for 2012: 1.2 in men and 1.8 in women in Goals, 1.00 and 1.7 in both Thembisa and EMOD. Earlier models also did not generally estimate incidence rates as high as the 2012 HSRC observation ^25^. Possibly the 2012 survey over-estimated incidence, by assuming a zero false-recent rate ^47^. If our models used incidence that is too high, they would over-estimate the impact and cost-effectiveness. Nevertheless, to refine models and MMC impact projections in future, the fitting to incidence measurements, including from the 2017 national survey ^3^ whose incidence estimates was not yet used for the current modelling, may merit reconsideration.

Men might adopt riskier behaviors after circumcision; such behavioral risk compensation would lead to overestimation of MMC impact. Studies in KwaZulu-Natal ^63, 64^ and Orange Farm ^65^ in South Africa, similar to studies in Zimbabwe ^66^, Kenya ^67^ and Uganda ^68^, found no evidence for changed risk behaviours after MMC. However, surveys in Cape Town found perceptions or expectations of MMC-related risk compensation in both men and women ^69– 71^. A further source of impact overestimation could be elevated risk during wound healing. There is limited published evidence for or against this in South Africa; however a study in uMgungundlovu in Kwa-Zulu Natal found that 29% of adolescent boys resumed sex during the six week healing period ^72^.

### Programmatic implications

To monitor progress towards targets and ascertain MMC coverage by district, it is important that all data are captured in the DHIS. Particular attention is needed to ensure that the MMC numbers conducted at community level and by General Practitioners contracted by provinces are captured. To this end, training in the National Department of Health Standard Operating Procedure for MMC data flow process is required for all partners performing MMCs regardless of the location ^73^.

These results illustrate that transitioning from MMC scale-up into a ‘maintenance phase’ at which coverage would stay constant at the target achieved implies a substantial reduction in annual MMC numbers after the target year. However, in countries like South Africa with very high HIV incidence the program target should probably rather be to increase MC coverage to higher than 80%.

It is concerning that in some provinces (most notably the Western Cape), there has been limited adoption of MMC. This may be related to cultural resistance to MMC among the Xhosa ^74^, one of the major ethnic groups in the Western Cape and Eastern Cape provinces. Alternatively, this may be a reflection of a lack of clear policy or resources. Action is required to address the low rates of MMC uptake in some provinces.

In conclusion, this analysis shows that the MMC program in South Africa has already had impact, and its health and economic benefits will grow significantly in the future. An investment in MMC programs now will provide substantial benefits in the long-term.

## Data availability

### Models

All three models are freely available. Goals is part of the Spectrum modeling program and can be downloaded at: https://www.avenirhealth.org/software-spectrum.php. The Thembisa model is available for download at: https://www.thembisa.org/downloads. The EMOD software can be downloaded from: https://www.idmod.org/docs/hiv/install-overview.html

### Underlying data

DRYAD: The impact of the program for medical male circumcision on HIV in South Africa: analysis using three epidemiological models. https://doi.org/10.5061/dryad.b8gtht7bp ^46^

This project contains the following underlying data:

- ExtendedDataFile2 Goals model detail results_06Dec2020.xlsx (Excel file with results from the Goals model including scenario descriptions, new infection, HIV prevalence, population size, people living with HIV (PLHIV), number of circumcisions (VMMC) and number of people on antiretroviral therapy (ART))
- ExtendedDataFile2 Thembisa model detail results 06Dec2020.xlsx (Excel file with results from the Goals model including scenario descriptions, new infection, HIV prevalence, population size, people living with HIV (PLHIV), number of circumcisions (VMMC) and number of people on antiretroviral therapy (ART))
- ExtendedDataFile2 EMOD model detail results 06Dec2020.xlsx (Excel file with results from the Goals model including scenario descriptions, new infection, HIV prevalence, population size, people living with HIV (PLHIV), number of circumcisions (VMMC) and number of people on antiretroviral therapy (ART))

### Extended data

DRYAD: The impact of the program for medical male circumcision on HIV in South Africa: analysis using three epidemiological models. https://doi.org/10.5061/dryad.b8gtht7bp ^46^

This project contains the following extended data:

- Extended_Data_File1_28Dec2020_ek.docx (Document with additional figures and tables)

Data are available under the terms of the Creative Commons Zero "No rights reserved" data waiver (CC0 1.0 Public domain dedication).

## Author information

Clarence Manuhwa was an Independent Consultant whilst working on this article, but is currently affiliated with FHI 360.
