# Supplementary material for: The impact of the program for medical male circumcision on HIV in South Africa: analysis using three epidemiological models
Source: Gates Open Res. 2021 Jan 25;5:15. [Version 1] doi: 10.12688/gatesopenres.13220.1 (PMC7878969; doi:10.12688/gatesopenres.13220.1)
Supplement: Peer review history

# Peer review: Reviewer response for version 1

**DOI:** 10.21956/gatesopenres.14439.r30364

**Published:** 2021-02-11

**Authors:** Michel Garenne, Alan Matthews

**Affiliations:** 1 MRC/Wits Rural Public Health and Health Transitions Research Unit, School of Public Health, University of the Witwatersrand, Johannesburg, South Africa; 2 School of Chemistry and Physics, University of KwaZulu-Natal, Durban, South Africa

I read with interest the paper by Eline Korenromp and colleagues on the possible impact of male circumcision on HIV in South Africa.

 I have no specific comment with respect to the study, to the model, to the writing and presentation of results. It is all well done. However, I think that the conclusions are misleading, since they do not match demographic evidence. The  authors claim to have demonstrated an impact, but they have only simulated a potential impact with their model. Current demographic evidence shows rather the opposite: the VMMC had no visible effect on HIV in South Africa (as is the case in Zambia).

 Therefore, I have prepared, with my South African colleague Alan Matthews, a comment, that we would like to attach here.

Is the work clearly and accurately presented and does it cite the current literature?

Yes

If applicable, is the statistical analysis and its interpretation appropriate?

Yes

Are all the source data underlying the results available to ensure full reproducibility?

Yes

Is the study design appropriate and is the work technically sound?

Yes

Are the conclusions drawn adequately supported by the results?

Partly

Are sufficient details of methods and analysis provided to allow replication by others?

Yes

Reviewer Expertise:

Health demography

We confirm that we have read this submission and believe that we have an appropriate level of expertise to confirm that it is of an acceptable scientific standard.

# Peer review: Reviewer response for version 1

**DOI:** 10.21956/gatesopenres.14439.r30288

**Published:** 2021-02-11

**Authors:** Brian G. Williams

**Affiliations:** 1 South African Centre for Epidemiological Modelling and Analysis (SACEMA), Stellenbosch University, Stellenbosch, South Africa; 2 Retired Visiting Research Fellow, Stellenbosch, South Africa

This is an interesting and important analysis of the impact of medical male circumcision (MMC) on the epidemic of HIV in South Africa. South Africa has had a severe epidemic of HIV. Traditional male circumcision (TMC) is common in South Africa but carries attendant risks of mutilation or death so the availability and effectiveness of MMC is of particular relevance and importance. With a thorough review of the relevant literature and the application of three different models based on all the available data the authors are able to show that MMC has already averted about one hundred thousand HIV infections and is likely to avert four to five times as many in the next ten years. The authors show that the cost of this programme is not insignificant, at about US$1,000 per infection averted, but this is considerably less than the cost of life-long treatment for HIV of an infected person. The study design is excellent and the work is technically sound with sufficient detail provided to allow replication. Retrospective studies of this kind are seldom published but remain very important to confirm earlier predications and to provide support for programme managers.

Is the work clearly and accurately presented and does it cite the current literature?

Yes

If applicable, is the statistical analysis and its interpretation appropriate?

Yes

Are all the source data underlying the results available to ensure full reproducibility?

Yes

Is the study design appropriate and is the work technically sound?

Yes

Are the conclusions drawn adequately supported by the results?

Yes

Are sufficient details of methods and analysis provided to allow replication by others?

Yes

Reviewer Expertise:

Physics, Chemistry, Mathematics, Epidemiology, Public Health, HIV, TB.

I confirm that I have read this submission and believe that I have an appropriate level of expertise to confirm that it is of an acceptable scientific standard.
